# Women and a New World

**Published:** 1920-12-25

**Authors:** 


### THE PUBLIC WELL-BEING.

### Women and a New World.

A special correspondent of one of the daily newspapers has been pursuing an interesting quest. He has been seeking to discover how woman, now that she has the vote, will best assist in building up a new world. His method of approach, which in the view of many may not have been the ideal method or a very hopeful one, was to interview all the "representative" and well-known women whom he could reach and to invite their opinions. He certainly obtained a mass of opinions sufficient to fill several columns of his newspaper, but they were so conflicting, and in some cases so vague, that he appears to have been even more bewildered at the end than he was at the beginning.

This is not altogether surprising. For our part we should like to quote as an expression of what seems after all to be the right point of view the remarks made in one of these interviews by Lady Aberdeen, for twenty-three years President of the International Council of Women, who not only believes that a woman's point of view exists, but insists that unless women bring a new spirit into public life they will fail to justify the claim they have made. She says:

> We based our demand for political suffrage on the plea that not only would justice thereby be done to one-half of the human race, but that a great new spiritual force would be realised for the world's service through the influence of women, whose instinctive desire is to create and protect life.

The last sentence seems to sum up the truth of the matter. The important thing about the entrance of women into public life is not, of course, that they should increase sex antagonism, but that they should cause it to disappear; not that they should form a women's party, and so take a limited view of the needs of the community at large, but that they should be, as women, of no party, and unite their ideals with those of men. There are clearly many essentially practical ways in which the power and influence of women can be effectively and usefully employed.

But in the minds of those who are responsible for the health of the nation, or who from purely disinterested motives devote their lives to the work, there is one outstanding branch of public service in which, above all others, the assistance of women is to be welcomed. "The instinctive desire of women," says Lady Aberdeen, "is to create and protect life." It is in this regard that the new co-operation of women is specially desirable, because they have the benefit of their own experience to contribute to the common stock of knowledge, and they bring to the work a fresh enthusiasm, a deeper humanity. Women so far may have done nothing wonderful on boards of guardians, on social welfare committees, on town councils, and other bodies, or, more recently, as magistrates; but what they are already doing is to focus public attention on questions of such vital interest to the race as the care of unfit children, the conditions of work in factories, and all those matters of deep importance which come under the heading of maternity and child welfare. Incidentally, women have brought their influence to bear heavily on the right side of the legislation, which provides that juvenile delinquents will now be dealt with, not in the atmosphere of the police courts, but in non-magisterial conditions, and with women to assist in the work of administering justice tempered with wisdom.

The disappearance of slumdom is another vital question in which women will have a bigger "say" than they have ever had in the past. "If a strike could ever be justified," said Sir Kingsley Wood, of the Ministry of Health, speaking in London the other day, "it is a strike of the women concerning the badly-planned, insanitary dwellings in which so many of them have to give birth to the citizens of the future." Now, instead of "striking," women can bend their energies in public, as well as in private, to the elimination of the slum, that costly evil which breeds consumptives by tens of thousands, and places a burden on the community running into millions a year.

